# Epigenetic Regulation in Schizophrenia: Focus on Methylation and Histone Modifications in Human Studies

**DOI:** 10.3390/genes15030272

**Published:** 2024-02-21

**Authors:** Natasha Delphin, Caitlin Aust, Lyn Griffiths, Francesca Fernandez

**Affiliations:** 1 School of Health and Behavioural Sciences, Faculty of Health Sciences, Australian Catholic University, 1100 Nudgee Rd, Banyo, QLD 4014, Australia; natashamaedelphin@gmail.com (N.D.);; 2 Centre for Genomics and Personalised Health, School of Biomedical Sciences, Queensland University of Technology, 60 Musk Ave, Kelvin Grove, QLD 4059, Australia; lyn.griffiths@qut.edu.au; 3 Healthy Brain and Mind Research Centre, Australian Catholic University, Melbourne, VIC 3000, Australia

**Keywords:** epigenetic, methylation, histone, schizophrenia, post-translational modification, genes

## Abstract

Despite extensive research over the last few decades, the etiology of schizophrenia (SZ) remains unclear. SZ is a pathological disorder that is highly debilitating and deeply affects the lifestyle and minds of those affected. Several factors (one or in combination) have been reported as contributors to SZ pathogenesis, including neurodevelopmental, environmental, genetic and epigenetic factors. Deoxyribonucleic acid (DNA) methylation and post-translational modification (PTM) of histone proteins are potentially contributing epigenetic processes involved in transcriptional activity, chromatin folding, cell division and apoptotic processes, and DNA damage and repair. After establishing a summary of epigenetic processes in the context of schizophrenia, this review aims to highlight the current understanding of the role of DNA methylation and histone PTMs in this disorder and their potential roles in schizophrenia pathophysiology and pathogenesis.

## 1. Introduction

Schizophrenia (SZ) is a severe neuropsychiatric disorder affecting 1% of the general population and ranking among the top 10 causes of disability in developed countries worldwide. Importantly, it is a major cause of suicide in youth populations; out of more than 30% of SZ sufferers attempting suicide, 5% will succeed [1]. The disorder is exceptionally difficult to diagnose at first, striking down seemingly healthy individuals, usually in the second and third decades of their life [1]. Patients suffering from SZ exhibit three types of symptoms, including “positive” (i.e., excessive types of behaviors including hallucinations and delusions), “negative” (i.e., including decreased interest and motivation, such as avolition and anhedonia), and cognitive, as defined by the Diagnostic and Statistical Manual of Mental Disorders (DSM-5) [2]. Despite significant progress in understanding the mechanisms underlying SZ pathophysiology and the management of its symptoms, this disorder remains an elusive and complex etiology believed to be due to a combination of environmental, genetic and epigenetic factors [3,4,5,6,7,8]. Twin studies in schizophrenic families have reported a high heritability of this disorder, with estimates varying between 60 to 80%, and a complex polygenic architecture [9]; environmental factors such as obstetric complications, maternal viral infections or malnutrition, drug and stress exposure, and childhood trauma may also contribute to an increased risk of developing SZ [10,11,12]. Delivery by emergency caesarean section and use of forceps along with low birth weight, pre-eclampsia and bleeding during pregnancy have been associated with susceptibility to developing SZ [2,3,4]. Other factors such as place and time of birth (late winter or spring), along with advanced parental age and maternal elevated inflammatory factors (high blood levels of C reactive protein and Interleukin 8) may also be SZ risk factors [5,6,7,8]. Exposure to one or several risk factors prenatally or early in life seems to impact normal brain developmental, supporting the theory that SZ is a neurodevelopmental disorder [9,10]. Disturbances to brain developmental in the early stages of life have long-lasting effects via genetic and epigenetic modifications, which can appear in adolescence and later in life [11,12]. Environmental conditions during upbringing such as city living, social isolation, and exposure to neurotoxins such as psychostimulants and cannabis can increase the risk of a psychotic episode and/or developing SZ [13,14,15,16,17,18,19]. In summary, exposure to one or several of the adverse environments mentioned above, particularly during a sensitive developmental period, may lead to epigenetic modifications or “molecular scarring” [20], with long-lasting effects affecting neurobiology in some individuals. In addition to environmental factors, differences between males and females in the age of onset, negative, and affective symptoms have been consistently reported in recent decades [13,14,15].

**Figure 1:**
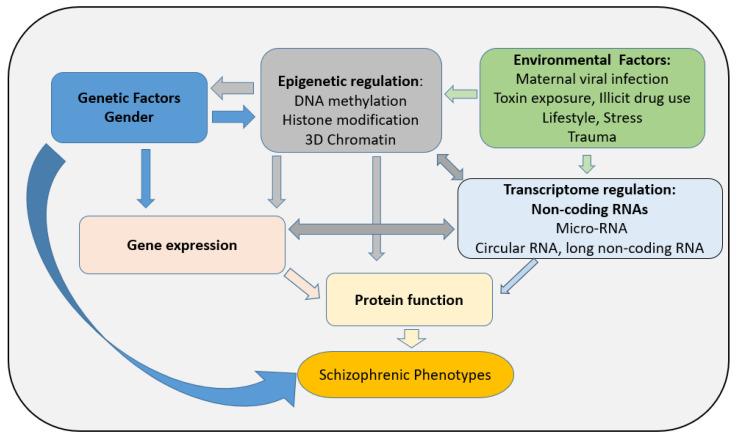
Factors influencing SZ pathogenesis: importance of epigenetic regulation.

Although the molecular mechanisms underpinning these sex differences and development of SZ later in life are still poorly understood, epigenetic regulation seems to play a critical role in the establishment of SZ and the development of pathology. In the last decade, epigenetic regulation has emerged as an integral component of brain development, and when dysfunctional may lead to some central nervous disorders including SZ [16,17,18]. Epigenetics is defined as alterations in gene expression without modification of the deoxyribonucleic acid (DNA) sequence [19]. Both DNA methylation and histone post-translational modifications (PTMs) constitute the main epigenetic regulators mediating the influence of the environment on the genome and regulating the cascade of transcriptional activity crucial for both the stability and plasticity of neuronal functioning (See Figure 1; [19,20]). Considering that SZ is known as a neurodevelopmental disorder, both genetic and environmental adverse conditions may lead to abnormal brain development with the symptoms of the disease appearing later in life. It is critical to further establish the role of epigenetic regulation in SZ, particularly in the context of brain plasticity and cognition. While transcriptome regulation including any type of non-coding RNA has been reported as an emergent regulator in epigenetic processes along with factors affecting chromatin remodeling complexes (such as the ATPase chromatin remodeler from the SNF2 superfamily of proteins [21]), these processes will not be reviewed here. In this review, we will focus on examining some of the current literature investigating changes in DNA methylation and PTMs in both central and peripheral tissues in SZ patients and discuss their implications for diagnosis and therapy for this pathology.

## 2. DNA Methylation: Focus on Genetic and Genomic Studies in SZ

DNA methylation is a mechanism of epigenetics that can regulate gene expression or suppression [22]. DNA methylation is catalyzed by DNA methyltransferases (DNMTs), which add a methyl group into fifth carbon position of the cytosine residue within the cytosine phosphate guanine (CpG) dinucleotide [23,24]. Gene expression or suppression are regulated by the DNMT family, including DNMT1, DNMT3a, DNMT3b, and DNMT3l [24]. While DNMT1 has been classified as the “maintenance methyltransferase” of the genome, DNMT3a and DNMT3b mediate de novo methylation [24]. In contrast, DNMT3l has no catalytic activity but can indirectly activate methylation via stimulation of DNMT3a and DNMT3b [25]. Active demethylation occurs via enzymes belonging to the ten-eleven translocation (TET) family proteins, undertaking thymine-DNA glycosylase (TDG)-mediated base excision repair for a return to unmethylated cytosines in DNA sequences [26].

Table 1 summarizes differentially methylated genes previously reported in postmortem tissues, blood, and saliva from SZ sufferers compared to controls. Cohort samples and methodology were also stated, but further conversion into 5hmC (hydroxymethyl-Cytosine) catalyzed by TET proteins has not been specifically addressed. The characterization of the presence of 5mC or 5hmC has been reported to be quite challenging since conventional bisulfite sequencing is unable to discriminate between these forms of modified cytosine [27]. For the majority of the studies a lower density of methylated CpG sites in the tested gene and/or the full genome in schizophrenia patients was noted compared to their respective control groups [28,29,30]. Interestingly, differential global hydroxymethylation levels were also found increased in male SZ patients, but decreased levels were found in female SZ patients compared to their respective controls [31]. Although only a limited number of studies have investigated global methylation in SZ, DNA methylation changes in SZ specific genes have been largely explored using a candidate gene strategy resulting from whole-genome approaches.

Recent findings have shown that dopamine (DA) hypothesis may be due to a specific DA dysregulation in SZ pathogenesis rather than overall DA hyperactivity involving selected receptor types and regional variations [32]. Levels of DA and its metabolites, homovanillic acid (HVA) and 3, 4-dihydroxyphenylacetic acid (DOPAC), were found significantly decreased in the cerebro-Spinal Fluid (CSF) from patients who stopped antipsychotic treatment [33,34]. Interestingly, numerous studies [35,36,37], but not all [38], have reported higher HVA levels in both CSF and plasma from schizophrenic patients who have acutely relapsed compared to stable patients. Plasma Catechol-O-methyltransferase (COMT), an enzyme involved in the metabolism of DA, is encoded by a gene in the 22q11.2 region, reported to have the strongest association with SZ in the largest genome-wide association studies (GWAS) of structural variations [39]. Hypomethylation of the promoter in the membrane-bound isoform of *COMT* (*MB-COMT*) gene was reported in 115 postmortem brain samples from the frontal lobe of SZ patients compared to healthy controls [40], a result not replicated in the frontal cortex in a smaller case–control cohort [41]. Interestingly, methylation profiling in a promoter of *DRD4*, *DRD5*, and *DRD2* genes was reported to be lower in blood from 80 SCZ cases compared to 71 healthy controls, with a significant differentially gene expression for *DRD2*, *DRD4*, and *DRD5* genes, but not for *DRD1* [42]. Taking into account that receptor targets of common antipsychotics (D2 receptors) and the variability of response to this treatment in SZ patients, DNA methylation may play an important part within disease development itself and/or response to treatment [43].

Recent findings suggest epigenetic mechanisms may also affect both the serotonin system and phenotypes induced by treatment with antipsychotics [23]. Interestingly, epigenetic dysregulation of both the *MB-COMT* and *5-HT2A* receptor in the brains of patients with SZ associated with an early age of disease onset was attenuated with anti-psychotic drugs [40,44]. Atypical antidepressants, such as mirtazapine and its structurally related counterpart mianserin, also block the function of 5-HT2A receptors. As mentioned above, hypermethylation was reported in the promoter of *5-HT2A* (-1438A/G polymorphism) in post-mortem frontal cortices of SZ patients compared with controls, thus affecting the response to antipsychotic medication [44]. Interestingly, reduction in serotoninergic transporter (*5-HTT*) gene expression was correlated with DNA hypermethylation in the *5-HTT* promoter in SZ patients (drug-naïve) when compared to controls, suggesting that an epigenetic controlled hypoactivity of 5-HTT neurotransmission may be linked to SZ pathogenesis [45].

In the same cerebral region, another hypermethylated promoter was reported for the glutamic acid decarboxylase 1 gene (*GAD1*), encoding for a rate-limiting enzyme converting glutamate into γ-aminobutyric acid (GABA) from SZ in postmortem tissue when compared to controls [1]. GAD dysfunction has been associated with changes in GABAergic circuitry, affecting a vast portfolio of functions including motor, cognitive, and behavioral functions [46], as reported in schizophrenic patients. Reelin (RELN), a glycoprotein mainly secreted by cells and a subpopulation of GABAergic interneurons, has also been reported as playing an essential role in the development of cortical neural connectivity in utero and synaptic plasticity at postnatal stages, both critical processes in SZ pathogenesis [47,48]. A significantly higher level of methylation in the *RELN* promoter was found in the SZ group compared to controls [49], significantly reducing *RELN* expression in different areas of the brain and blood in SZ individuals compared to healthy controls [50,51]. 

**Table 1:** Summary of DNA methylation from human tissues reported for candidate genes in SZ.

Pathways/Function	Genes	Tissues (n SZ vs. Controls C.)	DNA Methylation Status in SZ vs. Healthy Controls	Methodology	References
GABA and glutamate neurotransmission	*GAD 1* *NR3B*, *GRIA2* *GRM2*, *GRM5*	Frontal Cortex (5 SZ vs. 5 C.) Frontal Cortex (35 SZ vs. 35 C.) Blood (81 SZ vs. 71 C.)	Hypermethylation Hypomethylation Hypermethylation	Bisulfite sequencing methylation-specific PCR (MSP) Bisulfite sequencing Bisulfite sequencing and MSP	[1] [41] [52]
Dopaminergic neurotransmission	*MB-COMT* *DRD4*, *DRD5*, *DRD2*	Saliva (63 SZ vs. 76 C.) Dorsolateral Prefrontal cortex (PFC) (40 SZ vs. 40 C.) Blood (80 Sz vs. 81C.)	Hypomethylation Hypomethylation Hypomethylation	Bisulfite sequencing and MSP Bisulfite sequencing and MSP Bisulfite sequencing and MSP	[53] [40] [42]
Serotoninergic neurotransmission	*5-HT2A* *5-HT1A* *5-HTT*	Frontal Cortex (35 SZ vs.35 C.) Saliva (63 SZ vs. 76 C.) Saliva (40 SZ vs. 67 C.) Saliva/PFC (30 SZ vs. 20 C./ 35 SZ vs. 35 C.)	Hypermethylation Hypomethylation Hypermethylation Hypermethylation	Bisulfite sequencing and quantitativeMSP (qMSP) Bisulfite sequencing and qMSP Bisulfite sequencing and qMSP Bisulfite sequencing and qMSP	[44] [54] [55] [45]
Neuronal migration, dendrites, synaptogenesis and synaptic plasticity	*RELN*	Frontal Lobe (5 SZ vs. 5 C.) Occipital and PFC (15 SZ vs. 15 C.) PFC and Frontal cortex (14 SZ vs. 13 C./35 SZ vs. 35 C.)	Hypermethylation Hypermethylation No detectable difference	Bisulfite sequencing and qMSP Bisulfite sequencing and nested PCR Pyrosequencing Bisulfite sequencing	[50] [56] [41,57];
Neuronal growth and survival, synaptic plasticity	*BDNF*	PFC (17 SC vs. 17 C.)	Hypermethylation	Bisulfite sequencing and MSP	[58]
Embryonic development myelination	*SOX10* *LINGO-1*	PFC (11 SZ vs. 12 C.) Blood (268 SZ vs. 268 C.)	Hypermethylation Hypomethylation	Bisulfite sequencing Bisulfite sequencing and qMSP	[59] [60]
Transcriptional regulator in embryonic development Neuronal Growth	*FOXP2* *EGR1*	Para-hippocampus gyrus (13 SZ vs. 13 C.) Blood (64 SZ vs. 64 C.)	Hypermethylation No difference	Bisulfite sequencing Pyrosequencing	[27] [61]
Dendritic density Synaptic plasticity	*BAIAP2*	Superior temporal gyrus (16 SZ vs. 22 C.)	Hypomethylation	Bisulfite sequencing Genome methylation Bead Array	[62]

GAD 1: Glutamic acid decarboxylase 1, NR3: Nuclear Receptor Subfamily 3, DRD2: Dopamine Receptor D2, DRD4: Dopamine Receptor D4, DRD5: Dopamine Receptor D5, MB-COMT: Membrane-bound catechol-O-methyltransferase, 5-HT2C: 5-hydroxytryptamine-type receptor 2C, 5-HT1: 5-hydroxytryptamine-type 1 receptor, 5-HTT: 5-hydroxytryptamine transporter, GRM2: Glutamate Metabotropic Receptor 2, GRM5: Glutamate Metabotropic Receptor 5, GRIA2: Glutamate receptor ionotropic 2, RELN: Reelin, BDNF: Brain-derived neurotrophic factor, FOXP2: Forkhead box protein P2, BAIAP2: Brain-specific angiogenesis inhibitor 1-associated protein 2, LINGO-1: Leucine rich repeat and Immunoglobin-like domain-containing protein 1, EGR1: Early growth response r 1 and SOX10: SRY-related HMG-box 10.

Similarly to *RELN*, which is involved in synaptogenesis and synaptic plasticity in developing and adult brains, DNA methylation of additional genes essential for embryonic development (including *Brain-derived neurotrophic factor (BDNF)*, *Forkhead box protein P2 (FOXP2)*, *Brain-specific angiogenesis inhibitor 1-associated protein 2 (BAIAP2)*) and for the myelination process (such as *Leucine rich repeat and Immunoglobin-like domain-containing protein 1* (*LINGO-1)* and *SRY-related HMG-box 10 (SOX10)*) has been reported as differentially expressed in SZ-affected brain and blood tissues when compared to respective controls [27,58,59,60,61,62]. During development, DNA methylation in the genome is much more active than in a somatic adult cell, and this dynamic process involving both de novo DNA methylation and demethylation is critical for controlling gene expression and protein expression [24]. Hypermethylation has mostly been reported with decreased related gene expression [63]. As illustrated in Table 1, genes stimulating neuronal growth, differentiation and myelination and considered “positive” for healthy brain development for these processes were all reported to be hypermethylated (*BDNF*, *SOX10* and *FOXP2*) in SZ key brain structures (prefrontal cortex (PFC) and hippocampus) when compared to their respective controls, suggesting a decrease in gene expression of these positive factors in SZ. Meanwhile, a gene leading to inhibition of myelination (*LINGO-1*) and potentially considered a “negative’ factor in healthy brain development was reported as hypomethylated, suggesting a potential increase in expression of this gene [60,64]. Supporting this hypothesis, the LINGO-1 protein was reported to be significantly increased in post-mortem dorsolateral PFC in SZ when compared to controls [65]. Considering that the myelination process increases in a linear manner through infancy to middle-aged adulthood and taking into account hypomethylation of *LINGO-1* was associated with dysfunction of cognition function and white matter integrity in SZ when compared to controls [60], imbalance of methylation/demethylation patterns seems to persist and potentially increase across the lifetime of individuals suffering from schizophrenia.

Although variation of methylation profiling plays an important role in SZ development, future work is required to further characterize DNA methylation profiles expressed centrally and on the periphery in the context of SZ as well as their effects on related gene expression. DNA methylation can lead to either induced or suppressed gene expression depending on the region wherein it is situated (and other factors such as genetic vulnerability) [66]. Higher risk factors for SZ have been reported in individuals with a family history of psychosis who experienced a viral infection during fetal development compared to individuals with no reported infection [67], confirming the cumulative effects of both genetic and environmental factors. Over 1000 genes have been identified in SZ genetic susceptibility through association studies based on their chromosomal position and/or their function in SZ pathophysiology [68]. DNA methylation in these candidate genes (involved in critical neurotransmitter pathways such as dopamine or GABA) has been the focus of research for the last decade. As illustrated in Table 1, differences in methylation profiles have been reported for some candidate genes (for example, *RELN*) in the same tested region (PFC). This lack of reproducibility in DNA methylation studies [69] may be due to several limitations including cohort size and tissue quality [27], experimental protocol (pyrosequencing vs. bisulfite sequencing and quantitative methylation-specific PCR (qMSP) [70]), and other cofounders such as smoking [71], exercising [72] or use of medication (antipsychotics [73,74]). These limitations may also apply to other epigenetic process such as the methylation of proteins including histone proteins [69]. Modification of histone proteins has recently emerged as a critical post-translational change affecting gene expression via changes in chromatin structure.

## 3. Histone PTMs Roles in SZ Human Studies

### 3.1. Histone Proteins

Histone proteins are key structural units of chromosomes that mediate a higher level of folding of the chromatin [75]. The nucleosome contains an octamer of histones consisting of an H3-H4 tetramer and two H2A-H2B dimers [75]. In physiological conditions, the H3/H4 tetramer is the core of the histone octamer, and the H2A/H2B is symmetrically located on both sides of this tetramer. The nucleosomes are all joined by linker DNA (around 20 bp between each nucleosome) and histone H1 to form the chromatin. Binding to the nucleosomal core around the DNA entry and exit sites, the linker histone H1 can affect the stability of the nucleosome and chromatin architecture [76]. Slightly coiled chromatin presents DNA regions that allow transcription to occur, while tightly coiled chromatin comprises transcriptionally inactive DNA regions [77]. All histone proteins have a similar structure, which includes a globular domain and unstructured N-terminal ‘tail’ [76]. These histone tails do not contribute significantly to the structure of the nucleosomes, but they are essential for regulating chromatin’s degree of condensation into higher-order structures [78]

The N-terminal and C-terminal tails of H2A, H2B, H3 and H4 histones are subject to numerous and dynamic modifications. More than 70 histone amino acid modifications have been reported, including methylation, acetylation, phosphorylation, ubiquitination, and sumoylation [77]. Some of these histone modifications have been associated with transcriptional activation, for example, acetylation, while methylation controls gene activation and repression depending upon the specific position of the histone tail residue [77]. For instance, the methylation of histone H3meK5/K37 or K80 has been associated with actively transcribed genes, while methylation in H3meK10/K28 or K21 has been previously related to gene silencing [79]. Histone acetylation is performed by histone acetyltransferases (HATs), which catalyze the transfer of an acetyl group from acetyl Co-A to the lysine on the N-terminal tails of histone protein [80]. Acetylation can also be reversed through histone deacetylase (HDAC). The balance of these two dynamic processes is involved in the regulation of many cellular processes such as chromatin architecture, gene transcription, cell cycle and division, apoptosis, differentiation, and DNA replication and repair [78]. There are two main classes of HATs (A and B), which play an essential role in controlling H3 and H4 acetylation [81]. With opposite effects to HATs, HDAC enzymes wrap the DNA tightly around the histone proteins, leading to a decrease in gene transcription [82]. There are two types of HDACs families, sirtuin families and HDAC families, which include four sub-classes (1 to 4) [81]. Interestingly, the levels of HDAC1 in SZ were found to be increased when compared to controls in the PFC and hippocampus, brain structures essential for high functioning and cognition and both affected in SZ pathogenesis [83]. 

Considering the critical role of histone proteins in the fine-tuning and coordination of gene expressions on a spatiotemporal basis during neurogenesis, studies of histone proteins and PTMs are of significant interest for SZ pathogenesis due to the neurodevelopmental nature of this disorder. 

### 3.2. Role of Histone Modifications in SZ: Human Studies

Histone PTMs represent one of the epigenetic modulation switches determining the status of chromatin (restrictive vs. permissive) potentially involved in the pathogenesis of SZ, although there are a limited number of studies looking at the contribution of specific histone PTMs in SZ. Initial evidence originated from a report that valproate, a mood stabilizer, inhibits HDAC when administrated as a therapeutic [84]. Based on these observations, researchers have further explored whether SZ is associated with specific histone PTMs and/or alterations in the enzymes catalyzing such modifications. As illustrated in Table 2, the acetylation of H3 at lysine 9 and 14 (H3acK9/K14) is correlated with changes in the acetylation of promoters of SZ-related genes including *GAD67*, translocase of outer mitochondrial membrane 70 homolog A (*TOMM70A*), 5-hydroxytryptamine receptor 2C (*5-HT2C*), protein phosphatase 1E (*PPM1E*), and UDP-glycosyltransferase 8 (*UGT8*) and their levels of related levels of gene expression in young PFC postmortem SZ tissues only [85]. This finding is consistent with a previous study which reported common molecular changes in healthy human aging and the early stage of SZ [86]. The same acetylation of histone 3 H3acK9/K14, along with methylation (H3meR17, active chromatin) and phosphorylation at Serine 10 (H3pS10) were not reported associated with differential levels of gene expression in PFC, except for a subgroup of SZ exhibiting higher levels of methylation when compared to controls associated with changes in the level of expression of metabolic genes (see Table 2, [87]). It is unclear if the increased level of H3meR17 in the SZ group reflected an adaptive response to a decrease in the gene expression of metabolic genes specifically, or if it more generally reflected an alteration of the transcriptome in PFC in the SZ group. Interestingly, in the same brain region, the same histone 3 with 3 methylations was also associated with a decrease in *GAD 1* gene expression [1]. This finding is consistent with previous studies reporting a reduction of GAD gene expression associated with SZ in the frontal cortex and hippocampus, both brain structures highly involved in SZ pathophysiology [29,88,89,90].

Gene expression for *GAD 67* was also reported as being decreased in the PFC from 16 SZ when compared to 27 controls and correlated with an increased expression of *HDAC1, HDAC3* and *HDAC4* [91]. In contrast, relative HDAC1 expression was found lower in the dorsolateral PFC of patients with SCZ/SAD compared with controls, and interestingly, HDAC expression was also positively correlated with cognitive performance scores across groups [92]. This last study used a radiotracer version of the potent HDAC inhibitor [11C] Martinostat, which may explain the difference of results observed with postmortem brain studies [92]. Increased levels of HDAC 1 protein and mRNA were also reported significantly elevated in SZ in both the PFC and hippocampus when compared to controls [83]. In the same study, the levels of *HDAC* in blood samples were found to be higher in SZ patients who had encountered stress in their early life when compared with patients without this stressful experience at an early stage [83]. This finding is consistent with high levels of HDAC leading to a non-permissive chromatin and preventing transcription of genes involved in adult neurogenesis. Both HDAC 1 and 2 play a significant role in neocortex development, particularly for the control of the spatiotemporal neuron production, which is essential for the functional integrity of the brain structure [93]. Interestingly, during early brain development, HDAC1 and HDAC2 show an overlapping pattern of expression [90,94]. However, postnatally, contrasting patterns of expression for HDAC 1 and HDAC 2 were reported in brain [95]. In addition, the expression of HDAC1 is primarily in glial cells, while HDAC2 is predominantly expressed in mature neurons [95]. Imbalance of these expressions along with aberrant histone PTMs could be one of the major contributors to the development of neuropsychiatric diseases due to their critical role during neurodevelopment. Future longitudinal studies will be required to fully characterize the role of histone modification at different stages of development in SZ.

**Table 2:** Summary of histone proteins and histone PTMs in human tissues in SZ.

Histone Proteins	PTMs	Tissues	Human Cohort	Main Findings	References
H3acK9/K14	Acetylation	Postmortem prefrontal cortex (PFC)	32 SZ vs. 34 controls (C.)	Decreased genes expression levels of *GAD1*, *TOMM70A*, and *HT2C*, in young SZ groups, not old SZ groups when compared with controls	[85]
H3meR17 H3pS10 H3acK9/14	Methylation Phosphorylation Acetylation	Postmortem PFC	41 SZ vs. 41 C.	No significant difference, except for a subgroup of SZ (*n* = 6) with higher levels of methylation (H3meR17) when compared to controls associated with decreased of 3 metabolic transcripts *CRYM*, *CYTOC/CYC1* and *MDH.*	[87]
H3meK4	Trimethylation	Postmortem PFC	36 and 50 matched case-control cohorts for SZ	H3K4-trimethylation in SZ (predominantly in females) associated with decreased of *GAD1* gene expression	[96]
H3acK9/K14 H3S10	Acetylation Phosphorylation	PBMCs	Clinical population with SZ vs. healthy individuals	H3K9/K14ac levels were significantly lower in SZ cultured cells compared to controls	[97]
HDAC1 HDAC3 HDAC4		Postmortem PFC	16 SZ vs. 27 C.	*HDAC1* levels higher in SZ compared to controls. *GAD67* gene expression negatively correlated with mRNA levels for *HDAC1*, *3* and *4*	[91]
HDAC		Postmortem dorsolateral PFC	14 SZ or schizoaffective vs. 17 C.	HDAC levels significantly lower in SZ when compared to controls	[92]
HDAC 1 HDAC 2		Postmortem dorsolateral PFC	175 SZ vs. 210 C.	mRNA *HDAC2* levels were significantly lower in SZ compared to control group, no difference for *HDAC1* mRNA levels	[98]
HDAC		Postmortem PFC and hippocampus	10 SZ vs. 11 C.	HDAC 1 levels were higher in SZ group compared to controls	[83]

GAD 1: Glutamic acid decarboxylase 1, GAD 67: Glutamic acid decarboxylase 67, TOMM70A: translocase of outer mitochondrial membrane 70 homolog A, 5HT-2C: 5-hydroxytryptamine receptor 2C, PP1ME: protein phosphatase 1E and UGT8: UDP-glycosyltransferase 8, CRYM: NADP-regulated thyroid-hormone-binding protein, CYTOC/CYC1: cytochrome somatic C 1 and MDH: Malate dehydrogenase.

## 4. Discussion

There is a growing body of studies investigating epigenetic mechanisms potentially involved in neurodegenerative and neuropsychiatric conditions. Animal models, cell lines, postmortem brain studies, and/or clinical studies have all demonstrated a dysregulation of epigenetic processes in SZ. This review has reported major work performed using human tissues and highlighted the importance of epigenetic regulation, particularly during brain development (pre- and postnatally). Dysregulation of epigenetic regulation may lead to reprogramming key functional genes (such as *GAD*) and changing the course of healthy brain development. The result of these functional brain changes increases the risk of psychiatric disorders. Interestingly, epigenetic modifications are also involved in maintenance of sex differences in the brain [99], which may explain the differences in susceptibility, onset, pathogenesis, and severity of SZ between males and females [100]. SZ in women seems less severe, with a delayed onset and lower incidence compared to men [101]. Interestingly, global DNA methylation in men has been reported to be higher than in women [102], a process which may contribute to differences in SZ phenotypes between genders. Another study reported that SZ female patients display around twice the amount of HDAC1 levels that male patients do; however, this result must be considered with caution as only 3 females vs. 13 males were included in this study, thus greatly limiting its interpretation [91]. Peripheral studies have also reported a higher levels of histone methyltransferases mRNA (*G9 α*, *SETDB1* and *GLP*) and methylation of H3K9 in men when compared to women [100]. The high levels of histone methyltransferases observed in men were also associated with higher expression of SZ symptoms and poorer quality of life when compared to women [100]. These findings support a sex-dependent epigenome potentially contributing to SZ etiology and disease development. Although additional work is needed to establish a clear sex difference in epigenetic regulation, it is important to keep this in mind while developing potential therapeutic action through epigenome modulation.

As reported in Table 1 and Table 2, the majority of SZ epigenetic studies have been performed using peripheral samples [42,53] and/or postmortem brain tissues [29,41]. However, the use of postmortem tissues introduces limitations for co-founders (which may influence epigenetic regulation) such as drug use (alcohol, cannabis, tobacco, etc.), medication use, and cause of death [20,71]. For instance, tobacco use was reported to significantly affect global DNA methylation, leading to epigenome and transcriptome changes [103]. Consequently, it is difficult to clearly determine which epigenetic processes are solely specific to SZ. In the meantime, variations in SZ phenotypes may be due to epigenetic variations, taking into account these various co-founders, and life experiences; this reflects the variability of SZ symptoms and severity observed in SZ cohorts [20]. 

There is a growing body of literature studying the use of medication (antipsychotics) in the context of epigenetic processes [73,104,105,106]. In a longitudinal study, treatment with clozapine, an atypical antipsychotic drug, led to higher methylation levels correlated negatively with diagnosis [106]. Higher methylation levels were demonstrated in antipsychotic-treated patients, with haloperidol treatment reversing DNA methylation levels close to levels similar to healthy control groups [107]. Chronic treatment with clozapine and sulpiride, but not haloperidol and olanzapine, induced the demethylation of the methylated genes of *RELN* and *GAD67* (glutamic acid decarboxylase 67) reported in SZ patients, potentially leading to the restoration of GABAergic gene expression and neurotransmission in SZ brains [108,109]. Although clinical studies showed that antipsychotic treatment can alter the methylation patterns of SZ genes and related gene expression in SZ patients, it is important to consider that variation of methylation patterns prior to the use of SZ medication can also affect the influence of the efficacy of antipsychotics in patients [110]. More work in drug-naïve patients is necessary to further determine the role of antipsychotic drugs on DNA methylation.

Clinical studies have reported an upregulation of HDAC2 in the human frontal cortex after chronic administration of atypical antipsychotic drugs [111]. This finding was also associated with a 5HT2A-dependent regulation of *HDAC2* transcriptional activity and an increase in the binding of HDAC2 with the promoter region of the metabotropic glutamate 2 receptor *mGlu2* gene [111]. Previous clinical trials with mGlu receptor 2 agonist showed high efficacy in providing a therapeutic effect on SZ [112], potentially via normalization of thalamo-cortical glutamatergic neurotransmission in PFC. A decrease in histone acetylation at the *mGlu2* promoter due to upregulation of HDAC 2 leads to alteration of the chromatin state at the *mGlu2* promoter and consequently limits the effects of atypical antipsychotic drugs [111]. Consequently, use of HDAC 2 inhibitors may be a new avenue of therapy for SZ, particularly for patients resistant to common antipsychotic medications. Valproate administration (which acts as a nonspecific HDAC inhibitor) has been reported to improve the clinical efficacy of atypical antipsychotic drugs (such as clozapine, risperidone, and olanzapine) [113,114,115,116], confirming the potential of HDAC inhibitors as new targets for SZ treatment. In the last few years, HDAC inhibitors have been extensively tested for different types of cancer therapy [117,118,119] and as cognitive enhancers [120], which may potentially help neurodegenerative diseases such as Alzheimer’s disease [121]. The use of HDAC inhibitors seems to have limited side effects and act as a permissive chromatin, making it accessible to all genes implicated in learning and memory. The role of HDAC 2 in cognition has been extensively reported and may be a target of preference for cognitive disorders [122]. Further work including appropriate controlled populations (potentially in a drug-naïve cohort) will be required to fully assess the role of epigenetic regulation in SZ therapy. 

This review has focused on two main epigenetic regulation processes (DNA methylation and histone PTMs and enzymes) in the context of SZ, with results demonstrating variability according to tested tissues and/or techniques. A better understanding of the relationship between clinical heterogeneity and epigenetic profiling is necessary. Although several factors such as gender, age of onset and course of the disease, comorbidity with other disease (for example, depression), lifestyle, and use of medication can affect the epigenome in SZ, further studies are needed. To further understand the potential involvement of the epigenome in SZ development and/or pathophysiology, studies should control for these confounding factors, which can explain the variation in findings. It is essential to determine when epigenetically induced disease occurs (e.g., before SZ symptoms can be seen) and whether it could be a secondary effect of SZ pathophysiology and/or medication when treated [123,124]. Combining longitudinal epigenetic studies with genome-wide association (such as array-based platforms or next-generation sequencing) and SZ twin studies will allow us to gain a better understanding of both genetic and environmental effects on the epigenome in the context of SZ [125]. Bisulfite-modified whole-genome sequencing approaches have demonstrated DNA methylation analysis of base pair resolution [126]. In addition, differences between human sample types, tissues, and cell heterogeneity along with diverse techniques used to determine epigenetic marks and patterns should also be taken into account and controlled for when possible [69]. Discrimination between various cytosine modifications on a genome-wide scale and cell sorting-based analyses will help with identification of epigenetic profiling in diverse SZ tissues [127,128,129]. To further consider epigenetic markers as potential biomarkers for SZ, studies with specific cell types and defined tissues should be compared in both brain tissues and peripheral tissues (blood and saliva), with the common epigenetic pattern in both brain and peripheral tissues being a promising biomarker for SZ [12,29]. This review also highlights the promising but so far limited clinical application of pharmaco-epigenetics (regulating both DNA methylation and HDAC activity) to SZ due to the heterogeneity of findings across different cells, tissues, and populations. However, there is hope that epigenetic regulation will be considered when determining clinical therapeutic decisions for SZ sufferers, similar to what is seen in cancer therapy [130]. A future tailor-made therapy may be developed in relation to a patient’s epigenetic profile in order to provide the most effective way of treating SZ. Current preclinical studies investigating HDAC inhibitors look promising. Regulation of histone acetylation via pharmacological action on histone acetylation readers (bromodomain and extra-terminal (or BET)) at an early stage of the disease may offer early therapeutic intervention for the disease [125]. 

In summary, since epigenetic modification may be associated with treatments for disease, they may also act as predictors pf treatment response [131] and/or targets for future therapy. Acting on epigenetic regulation may reinstate gene expression activation due to chromatin status loss during neurodevelopment in SZ pathogenesis and restore previously constrained or dormant neurotransmission, which is affected by epigenetic factors and/or environmental stress. Future studies including drugs targeting epigenetic regulators will help to establish future avenues of therapy for SZ.

## Data Availability

Not applicable.
